# Differential Susceptibility of Ex Vivo Primary Glioblastoma Tumors to Oncolytic Effect of Modified Zika Virus

**DOI:** 10.3390/cells12192384

**Published:** 2023-09-29

**Authors:** Gustavo Garcia, Nikhil Chakravarty, Sophia Paiola, Estrella Urena, Priya Gyani, Christopher Tse, Samuel W. French, Moise Danielpour, Joshua J. Breunig, David A. Nathanson, Vaithilingaraja Arumugaswami

**Affiliations:** 1 Department of Molecular and Medical Pharmacology, University of California Los Angeles, Los Angeles, CA 90095, USA; gustavogarcia@berkeley.edu (G.G.J.); dnathanson@mednet.ucla.edu (D.A.N.); 2 Department of Epidemiology, University of California Los Angeles, Los Angeles, CA 90095, USA; nikhilc23@ucla.edu; 3 Department of Pathology and Laboratory Medicine, University of California Los Angeles, Los Angeles, CA 90095, USA; sfrench@mednet.ucla.edu; 4 Jonsson Comprehensive Cancer Center, University of California Los Angeles, Los Angeles, CA 90095, USA; 5 Department of Neurosurgery, Cedars-Sinai Medical Center, Los Angeles, CA 90048, USA; moise.danielpour@cshs.org (M.D.); joshua.breunig@cshs.org (J.J.B.); 6 Board of Governors Regenerative Medicine Institute, Cedars-Sinai Medical Center, Los Angeles, CA 90048, USA; 7 Department of Biomedical Sciences, Cedars-Sinai Medical Center, Los Angeles, CA 90048, USA; 8 Department of Medicine, David Geffen School of Medicine, University of California Los Angeles, Los Angeles, CA 90095, USA; 9 Eli and Edythe Broad Center of Regenerative Medicine and Stem Cell Research, University of California Los Angeles, Los Angeles, CA 90095, USA; 10 California NanoSystems Institute, University of California Los Angeles, Los Angeles, CA 90095, USA

**Keywords:** Zika virus, glioblastoma, oncolytic virus, apoptosis, neurosphere

## Abstract

Glioblastoma (GBM), the most common primary malignant brain tumor, is a highly lethal form of cancer with a very limited set of treatment options. High heterogeneity in the tumor cell population and the invasive nature of these cells decrease the likely efficacy of traditional cancer treatments, thus requiring research into novel treatment options. The use of oncolytic viruses as potential therapeutics has been researched for some time. Zika virus (ZIKV) has demonstrated oncotropism and oncolytic effects on GBM stem cells (GSCs). To address the need for safe and effective GBM treatments, we designed an attenuated ZIKV strain (ZOL-1) that does not cause paralytic or neurological diseases in mouse models compared with unmodified ZIKV. Importantly, we found that patient-derived GBM tumors exhibited susceptibility (responders) and non-susceptibility (non-responders) to ZOL-1-mediated tumor cell killing, as evidenced by differential apoptotic cell death and cell viability upon ZOL-1 treatment. The oncolytic effect observed in responder cells was seen both in vitro in neurosphere models and in vivo upon xenograft. Finally, we observed that the use of ZOL-1 as combination therapy with multiple PI3K-AKT inhibitors in non-responder GBM resulted in enhanced chemotherapeutic efficacy. Altogether, this study establishes ZOL-1 as a safe and effective treatment against GBM and provides a foundation to conduct further studies evaluating its potential as an effective adjuvant with other chemotherapies and kinase inhibitors.

## 1. Introduction

Cerebral gliomas are the most common primary brain tumors, accounting for 80% of all primary pediatric and adult non-metastatic malignant brain tumors [1]. Glioblastoma (GBM), the most common primary malignant brain tumor [1], is nearly universally fatal, with median survival under two years [2]. Despite recent advancements in research, only modest progress has been made in achieving glioma patient prognosis and quality of life [3]. GBM comprises a heterogeneous cell population, consisting of cancer stem cells (CSCs) and varying degrees of more differentiated tumor cells [4]. GBM likely arises from transformed glial populations of oligodendrocytes [5,6]. The tumor-propagating cells are a mix of oligodendrocyte progenitor cells, astrocytes, and undifferentiated glioblasts [5,6]. The rapid-growing nature of GBM can impact the surrounding brain tissue, increasing intracranial pressure, causing patients to experience symptoms such as severe headaches, nausea, vomiting, drowsiness, and focal or progressive neurological deficits [7]. Beyond these general symptoms, patients can develop symptoms dependent on the location of the GBM in the brain, such as general body weakness; seizures; difficulties in speech, memory and cognition; and hearing and vision impairments [8]. Despite some new developments in treatments for GBM, patient survival is still unfortunately low, with high rates of recurrence. The main conclusion of GBM treatment has been that a single-target approach is not effective [9]. As such, there is an urgent need for research into novel, effective treatments.

The use of oncolytic viruses for the treatment of GBM has been investigated, with measles virus [10,11,12,13], poliovirus [14,15], adenovirus [16,17], herpesviruses [18,19,20,21], myxoma virus [22,23], vesicular stomatitis virus [24], reovirus [25], parvovirus [26], and rhinovirus [27] having been investigated in laboratory in vitro and in vivo studies, as well as in several clinical trials. As of September 2023, there were 57 completed public clinical trials testing oncolytic viruses [28], with outcomes indicating a positive response by patients to treatment delivery with some antitumor effects. At present, a modified herpesvirus and a reovirus have received US Food and Drug Administration (FDA) approval for cancer treatment [29], with the reovirus treatment (pelareorep) receiving orphan drug designation for the treatment of gastric cancer [30].

The Zika virus (ZIKV) is transmitted by *Aedes* mosquitoes and was first discovered in 1947, carried by rhesus macaque monkeys in Uganda [31]. The virus has since spread across the world, with infections reported in Asia, the Americas, the Caribbean, the Pacific Islands, and Mexico [32]. The 2015-16 outbreak in South America and the Caribbean attracted global attention due to the dire teratogenic effects caused by ZIKV infection, including microcephaly and fetal mortality in infants born to infected mothers [33,34]. Other severe symptoms of the immune and central nervous systems, such as Guillain–Barré syndrome, encephalitis, myelitis, and acute disseminated encephalomyelitis, can also manifest as a result of Zika disease [33,34].

Because fetal neuroprogenitor (or neuroblast) cells are the target of ZIKV replication as opposed to adult brain cells [35], we hypothesized that ZIKV may exert an oncolytic effect on GSCs, which share properties with fetal neuroprogenitor cells [36,37,38,39,40]. ZIKV can stimulate antitumor immunity as it lyses cancer cells, promoting the release of interferons (IFNs), chemokines, Toll-like receptor (TLR) agonists, tumor-associated antigens, danger-associated molecular patterns (DAMPs), and pathogen-associated molecular patterns (PAMPs) [41]. The release of these molecules can change the tumor microenvironment from its normally immunosuppressive nature to a more pro-immunogenic environment, allowing for immune cell infiltration and priming potentially long-term adaptive immune response against the virus and tumor cells [42]. We and others have demonstrated the oncolytic potential of ZIKV in GSCs [41,43,44,45,46,47,48]. Combining various oncolytic viruses with other anticancer therapeutics such as immunotherapies [42], chemotherapies [49], and radiotherapies [50] has been shown to enhance anticancer effects. Together, these findings have spurred active investigation into the utilization of modified oncolytic ZIKV strains as a personalized medicine approach to treating GBM. Questions remain regarding the potential of oncolytic ZIKV stimulating systemic efficacy and antitumor immunity. Few serious adverse events have been reported in human trials of oncolytic viruses [51]. However, in order to have effective antitumor activity, the virus should have sufficient attenuation to not cause severe disease but still maintain active replication in the tumor cells to cause tumor cell death. There are also questions regarding safety, given the potential for severe ZIKV-associated symptoms, as virulence can be a concern if improperly attenuated or wild-type ZIKV is used as an oncolytic agent. Using an attenuated mutation in the Envelope protein, we address the concern of virulence by preventing aggressive virus replication and spread. We further demonstrate differential responses to our modified oncolytic ZIKV, which induced therapeutic responses in human-derived GBM cells. Together, this investigation highlights a system that can be utilized to examine potential responses of different cancer types prior to treatment initiation.

## 2. Materials and Methods

### 2.1. Ethics Statement

This study was performed in strict accordance with the recommendations of the Guide for the Care and Use of Laboratory Animals. The institutional Animal Care Use Committee of UCLA approved the study. All patient tissue used to derive GBM cell cultures was obtained after obtaining explicit informed consent, and its use was approved by the UCLA Institutional Review Board (IRB) (IRB#10-000655, approved 23 February 2023).

### 2.2. Cell Lines

The human glioblastoma cell line U-87 MG and the Vero cell line were purchased from ATCC (Manassas, VA, USA). Cells were incubated at 37 °C and supplemented with 5% CO_2_. They were then subcultured when 90% confluence was reached (approximately every two-to-three days) using 0.05% trypsin plus 0.53 mM EDTA (Corning, Corning, NY, USA). GBM neurospheres were established and maintained as previously described [52]. Briefly, cells were cultured DMEM/F12 (Gibco, New York, NY, USA), B27 (Invitrogen, Waltham, MA, USA), penicillin–streptomycin (Invitrogen), GlutaMAX (Invitrogen) supplemented with heparin (5 μg/mL; Sigma, St. Louis, MO, USA), EGF (20 ng/mL; Sigma), and FGF (20 ng/mL; Sigma). All cells were grown at 37 °C, 20% O_2_, and 5% CO_2_; were routinely monitored; and tested negative for the presence of mycoplasma with a commercially available kit (MycoAlert, Lonza, Basel, Switzerland).

### 2.3. Zika Virus

The PRVABC59 (GenBank accession number KU501215) Zika virus strain of Asian genotype was used for the infection. PRVABC59 was acquired from the CDC, USA. Early-passage African genotype MR-766 ZIKV stock was acquired from ATCC. ZOL-1 recombinant virus was previously generated by our group [53]. Working viral stock for the specified experiments was generated by subjecting the original ZIKV strain (passage = 3) to two additional passages in Vero cells. An established viral plaque assay was utilized to measure viral titer as previously described [54].

### 2.4. Zika Viral Infection

For ZIKV infection, GBM cells were seeded in a 96-well plate at a cell density of approximately 1 × 10^4^ cells/well. Twenty-four hours after plating, ZIKV inoculum (MOI 1 or 5) was formulated using the medium specified above for each cell type. A volume of 100 µL of viral inoculum was added to each well. Plates were incubated at 37 °C with 5% CO_2_ for 2-to-4 h. After incubation, the medium was replaced for each cell type with cell-type-specific complete medium at a volume of 200 µL/well. For the uninfected (mock) group, each cell type received the specified cell growth medium used to prepare the viral inoculum as described above. A mock-infected control was used for each cell type at each specified timepoint of infection. Cell culture supernatants were harvested for viral titer analysis at each timepoint.

### 2.5. Quantification of ZIKV with Plaque Assay

Vero cells (ATCC) were plated on 48-well plates. Virus supernatants from infected cells or serum were titered with serial dilutions in DMEM. Titered supernatant was then added to Vero cell monolayers at 37 °C for 4 h. Fresh complete medium was then added. Two days after infection, plaques were counted as previously described [35].

### 2.6. Mouse Experiment

*Ifnar1^−/−^* (IFN-αβR-KO) mice (Jackson Laboratory, Bar Harbor, ME, USA; MMRRC Stock No. 32045-JAX) were used (n = 6–9 per group). The mouse is the most commonly used small-animal model system to study ZIKV pathogenesis in vivo, better allowing us to study ZIKV-mediated brain disease. A recent study by the Arumugaswami lab [41] has shown that direct inoculation of ZIKV in *Ifnar1^−/−^* mice results in brain infection similar to that seen in human patients. We thus continue to use this mouse model for ZIKV infection studies. Mice were housed at UCLA Division of Laboratory Animal Medicine (DLAM). Four–six-week-old *Ifnar1^−/−^* male and female mice were inoculated with phosphate-buffered saline (PBS) (n = 6 mice), PRVABC59 ZIKV (1 × 10^6^ pfu per mouse in a 40 µL volume), MR-766 ZIKV (1 × 10^6^ pfu per mouse in a 40 µL volume) (n = 6 mice), or ZOL-1 via the subcutaneous route in the hind limb region under isoflurane anesthesia. Blood samples were collected 7 dpi for measuring serum virus titer.

### 2.7. Mouse GBM Xenograft Study

GBM tumor cells were cultured as specified above. We used 6-week-old female NSG mice (n = 8 per group). Cells were mixed with Matrigel and then xenografted into *NSG* mice (1 × 10^6^ GBM cells/animal) via the subcutaneous route in the left-flank region. For the ZOL-1 pre-treatment group, U87 cells were infected with ZOL-1 (MOI 1) 2 h prior to subcutaneous implantation. The mice were monitored daily for tumor development. Once palpable mass was detected, the tumor volume was measured. On indicated days, ZOL-1 or PBS was intratumorally administered. Mice with tumors showing signs of ulceration or reaching the volume of 2.5 cm^2^ were euthanized. At the endpoint, tumor tissues and blood serum were collected from all the experimental animals.

### 2.8. Immunohistochemistry

Tumor tissues were incubated in 4% PFA for 1 h and then transferred to PBS. Tissues were subsequently submerged in the following for one hour each: 10%, 20%, and 30% sucrose. Tissues were next embedded in OCT (Fisher Healthcare, Hampton, NH, USA) and incubated at −80 °C overnight. Tissues were cut in 6 μm thick slices using a Leica cryostat microtome and mounted on Super Frost microscope slides (VWR, Radnor, PA, USA). Sections were then washed 3 times and permeabilized using a blocking buffer (0.3% Triton X-100, 2% BSA, 5% Goat Serum, 5% Donkey Serum in 1× PBS) for 1 h at room temperature. For immunostaining, sections were incubated overnight at 4 °C with each primary antibody (Table S1). The sections were then rinsed with 1× PBS three times and incubated with the respective secondary antibody (Table S1) for 1 h at room temperature. DAPI (4′,6-diamidino-2-phenylindole, dihydrochloride) (Life Technologies, Carlsbad, CA, USA) was used to stain cell nuclei at 1:5000 dilution in blocking buffer. Image acquisition was performed using a Leica DM IL LED fluorescent microscope with Leica Application Suite X (LAS X).

### 2.9. Western Blot Analysis

Cells were lysed using lysis buffer (50 mM Tris (pH 7.4), 1% NP-40, 0.25% sodium deoxycholate, 1 mM EDTA, 150 mM NaCl, 1 mM Na3VO4, 20 Mm or NaF, 1 mM PMSF, 2 mg mL^−1^ aprotinin, 2 mg mL^−1^ leupeptin, and 0.7 mg mL^−1^ pepstatin or Laemmli Sample Buffer) (Bio Rad, Hercules, CA, USA). We resolved cell lysates by using 10% SDS-PAGE pre-cast gradient gels (Bio-Rad) and transferred to a 0.2 µm PVDF membrane utilizing the Trans-Bolt turbo transfer system (Bio-Rad). After transfer, membranes were blocked using 5% skim milk and 0.1% Tween-20 for 1 h at room temperature. Membranes were then probed with respective monoclonal antibodies (Table S1) and detected using the SuperSignal West Pico Chemiluminescent Substrate kit (Thermo Scientific, Waltham, MA, USA).

### 2.10. Data Analysis

All statistical testing was performed at the two-sided alpha level of 0.05. Data were analyzed for statistical significance using an unpaired Student’s *t*-test to compare the two groups (uninfected vs. infected) with Graph Pad Prism software, version 8.1.2 (GraphPad Software, La Jolla, CA, USA).

### 2.11. Data Availability

All relevant data regarding this manuscript are available from the above-listed authors.

## 3. Results

### 3.1. Attenuated ZIKV Strain ZOL-1 Targets GSCs and Is Safe in Mice

**Figure 1:**
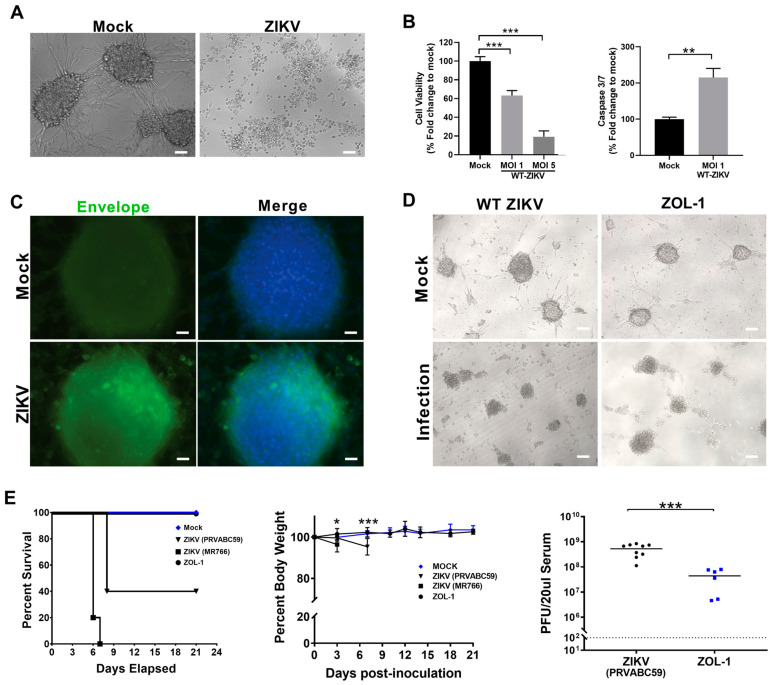
ZOL-1 replication in neurospheres and mice. (**A**) Bright-field images of ZIKV-infected (MOI 5) and uninfected (mock) U87 GBM cancer cells. (Scale bar: 50 μm.) (**B**) Cell viability and apoptosis. (**C**) Fluorescent microscopy images of mock- and ZIKV-infected (MOI 1) U87 glioma neurosphere cultures 2 dpi. A pan-flaviviral Envelope antibody was used to detect ZIKV-infected cells. (Scale bar: 25 μm.) Green = ZIKV Envelope protein; blue = DAPI. (**D**) Bright-field images of mock-, WT ZIKV (MOI 1)-, and ZOL-1 (MOI 1)-infected GBM spheroids 6 dpi. (Scale bar: 100 µm.) (**E**) Kaplan–Meier survival plot shows percent survival of *Ifnar1^−/−^* mice after inoculation with wild-type ZIKV (Asian and African strains) or ZOL-1 (n = 6 per group). Mean percent body weight change of mice after subcutaneous inoculation with mock, wild-type ZIKV, or ZOL-1 viruses. Serum viral load of ZOL-1 in inoculated mice 7 dpi. Dotted line indicates detection limit. Error bars represent the standard deviation. Student’s *t*-test: *p*-value < 0.05 (*), *p* < 0.001 (**), *p* < 0.0001 (***).

We initially tested the effect of wild-type (WT) ZIKV on the GBM cell line U87 and observed that U87 3D neurospheres [55] were highly susceptible to ZIKV infection and cell killing (Figure 1). ZIKV infection resulted in the near-complete disruption of the neurosphere structure, with primarily cell debris being seen (Figure 1A). Cell viability significantly decreased upon infection with ZIKV both at MOI 1 and MOI 5, with significantly increased levels of caspase 3/7, a marker of apoptotic cell death, upon MOI 1 ZIKV infection (Figure 1B). We then confirmed that these U87 cells were infected with WT ZIKV using fluorescent microscopy, marking for the ZIKV Envelope protein (Figure 1C). We found presence of the Envelope protein in U87 cells, confirming infection of these cells and resultant cell killing. This targeted killing effect by ZIKV on GSCs and other GBM cells has been substantiated by the existing literature [41,43,44,45,46,47]. Therefore, we generated an attenuated ZIKV oncolytic (ZOL-1) virus through a point mutation in the Envelope region (N154T) by removing the Envelope glycosylation site [53,56]. ZOL-1 still maintained antitumor activity, as was apparent through visible cell death (Figure 1D).

To assess the safety profile of ZOL-1, 4-to-6-week-old *Ifnar1^−/−^* mice (n = 6–9) were inoculated with either ZOL-1 (1 × 10^6^ PFU/mouse; subcutaneous route) or unmodified WT ZIKV (PRVABC59 and MR766 strains; positive control). The *Ifnar1^−/−^* model is the most commonly used small-animal model system for the evaluation of ZIKV pathogenesis in vivo [57]. As such, we utilized this model to better study ZIKV-mediated brain infection. Infected animals were monitored twice daily for three weeks. We observed that WT-ZIKV-infected animals showed significant mortality and exhibited signs of neurological disease, as evidenced by paralysis. Mock-infected and ZOL-1-inoculated mice exhibited 100% survival over the entire study period (Figure 1E). Beyond this, the serum viral load of ZOL-1 was 10- to 100-fold lower than that of WT ZIKV 7 days post-infection (dpi) (Figure 1E), indicating successful attenuation of the virus. Furthermore, mice infected with ZOL-1 stayed healthy over the course of the study, with no weight loss being observed (Figure 1E), suggesting a loss of ZIKV virulence upon attenuation.

### 3.2. GBM Cell Lines Respond Differently to ZOL-1 Infection

**Figure 2:**
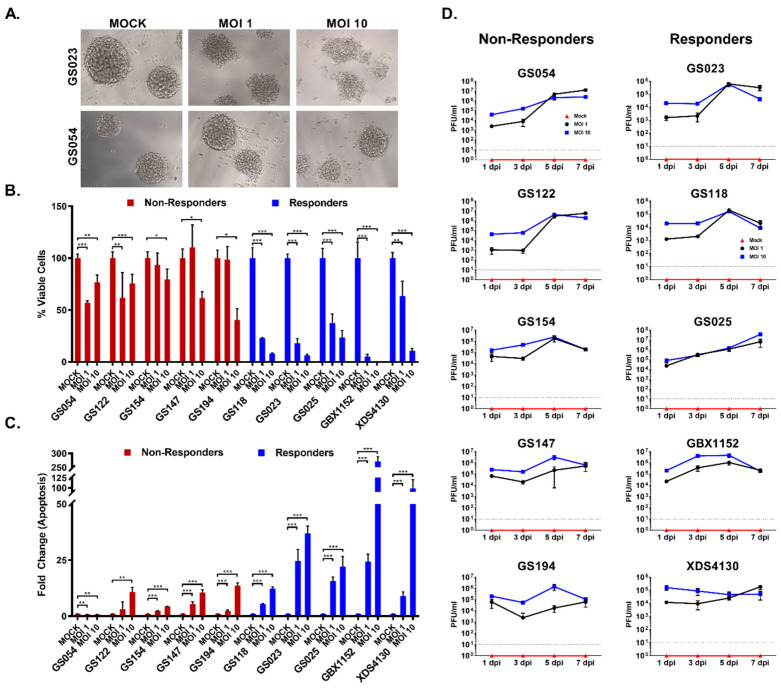
ZOL-1-mediated cell killing of patient-derived GBM tumor lines. (**A**) Bright-field images of ZOL-1-infected GBM responder (GS023) and non-responder (GS054) lines 7 dpi. (**B**) Cell viability of responder and non-responder tumor lines upon inoculation with ZOL-1 (MOI 1 or 10) or mock inoculation. (**C**) Apoptotic cell death, as measured using caspase 3/7 activity, of responder and non-responder tumor lines upon inoculation with ZOL-1 (MOI 1 or 10) or mock inoculation. (**D**) Viral titer of ZOL-1 in various GBM non-responders (left) and responders (right). Dotted line indicates detection limit. Error bars represent the standard deviation. Student’s *t*-test: *p*-value < 0.05 (*), *p* < 0.001 (**), *p* < 0.0001 (***).

GBM tumors are notoriously composed of a highly heterogeneous cell population with high plasticity [58]. As such, we set out to determine how this intratumoral heterogeneity may impact response to ZOL-1. To achieve this, we analyzed primary human GBM neurospheres from the UCLA GBM biobank that have been extensively well characterized based on their genotype and potential for engraftment in animal models. The cell types and their donor lineages can be found in Table 1. The tumor samples came from a racially diverse group of patients diagnosed with WHO grade IV GBM ranging from 39 to 74 years of age. Most tumors were recurrent, though two (GS023 and GS152) were newly diagnosed. GBX1152 and XDS4130 were established by xenografting human GBM lines GS152 and GS130, respectively, into mice. We infected each of these neurospheres with ZOL-1 at either MOI 1 or 10. After infection, two categories of responses were determined based on the levels of cell viability and apoptotic cell death observed (Figure 2A and Supplemental Figure S1B). We defined responders as those tumor types that showed an overall reduction in cell viability of less than 50% upon ZOL-1 treatment. The responder group (GS118, GS023, GS025, GBX1152, and XDS4130) displayed decreased cell viability and increased apoptosis, as evidenced by elevated levels of cleaved caspase 3 (Figure 2B,C). We note that XDS4130 is a borderline responder due to a robust decline in cell viability at MOI 10 treatment, despite cell viability being slightly above 50% at MOI 1. The non-responder group (GS054, GS122, GS147, GS194, GS194) was indifferent to viral infection, showing only minimal apoptotic activity (Figure 2B,C). Non-responder lines were predominantly found to have genetic changes in PTEN and EGFR (Table 1). In responder cell lines, we observed a dose-dependent decrease in cell viability corresponding with the increase in the level of ZOL-1 treatment (Figure 2B). Similarly, a positive dose-dependent interaction was also observed between the increase in dose and the increase in apoptosis levels (Figure 2C). Interestingly, we found that upon ZOL-1 treatment, viral titer and active replication were elevated in both responder and non-responder lines (Figure 2D). Seven days post-infection (dpi), we observed similarly elevated levels of viral replication regardless of dosage, suggesting viral affinity for GBM cells regardless of their treatment response status. Taken together, we propose that heterogeneous cancer cell lines, even those including the same cancer type, respond differently to treatment. This has important treatment implications given the heterogenous nature of naturally occurring tumors, as some patients with tumors containing these responder cell types may respond well to treatment with ZOL-1.

### 3.3. ZOL-1 Displays Clear Antitumor Effects on Xenograft NSG Mice

Having established differential in vitro susceptibility of GBM tumors to ZOL-1, we next evaluated the safety and efficacy properties of ZOL-1 in a GBM tumor cell-xenografted mouse model.

**Figure 3:**
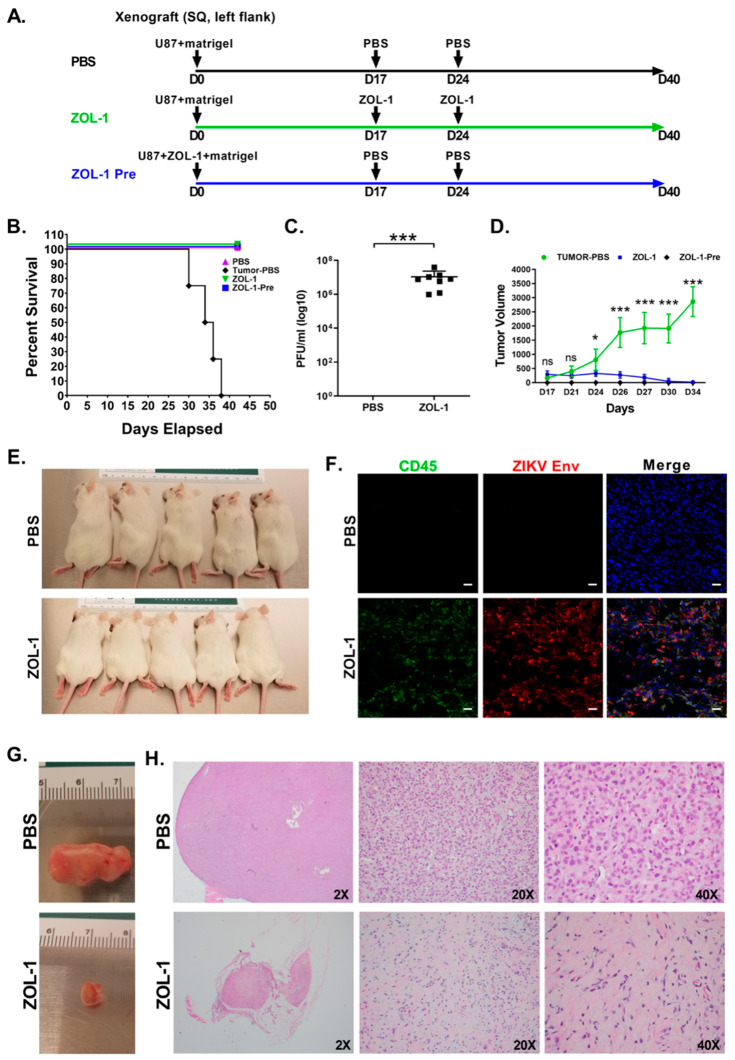
ZOL-1 efficacy against tumor xenograft in NSG mice. (**A**) Experimental design of GBM xenograft study with ZOL-1 treatment (n = 8 per group). (**B**) Kaplan–Meier survival plot shows percent survival of NSG mice upon mock treatment, or pre-treatment or treatment with ZOL-1. (**C**) Viral load of ZOL-1-treated mice at study endpoint (42 dpi). (**D**) Tumor volume measured post-xenograft injection over time. (**E**) Representative pictures of tumors in mock-treated and ZOL-1-treated mice show reduction in tumor size upon treatment with ZOL-1. (**F**) Immunofluorescence images show CD45 and ZIKV Envelope protein 7 dpi. (**G**) Representative images of resected tumors from mock-treated (top) and ZOL-1-treated (bottom) mice. (**H**) Histopathological analysis of ZOL-1-treated tumors compared with mock-treated tumors (image magnifications are provided). Error bars represent the standard deviation. Student’s *t*-test: *p*-value not significant (ns), *p* < 0.05 (*), *p* < 0.0001 (***).

Accordingly, NSG mice (n = 8 per group) were xenografted with U87 cells embedded with Matrigel in the left-flank region. A schematic of the study timeline can be found in Figure 3A. Mice receiving ZOL-1 pre-treatment were xenografted with U87 cells infected with ZOL-1. Animals in this group did not develop tumors, whereas animals receiving U87 cells alone developed visible and palpable tumor masses at the inoculated site by day 17. Subsequently, the tumor-containing mice received the first dose of ZOL-1 (2 × 10^6^ PFU/mouse) or PBS (mock-treated; negative control) intratumorally and the second dose one week later. We observed that the PBS-treated group succumbed to cancer burden before day 40, whereas those treated with ZOL-1 underwent tumor remission (Figure 3B). We were able to recover virus from ZOL-1-treated mice (Figure 3C), indicating that the observed antitumor effect was due to the direct oncolytic effect of the virus. Similarly, while observing significant increases in tumor volume in the mock-treated group, we found significant decreases and near eradication of the tumor mass in mice treated with ZOL-1 (Figure 3D), with ZOL-1 treatment showing visible decreases in tumor size (Figure 3E). Immunohistochemical analysis showed viral replication in tumor cells 7 days post-treatment, as evidenced by the presence of the ZIKV Envelope protein, as well as infiltration of CD45^+^ inflammatory cells and apoptotic cell death (Figure 3F and Supplementary Figure S2). ZOL-1-mediated tumor killing recruited inflammatory cell infiltration, whereas untreated tumors remained “cold”, with none-to-minimal inflammatory cells. The analysis of gross and histopathological images of PBS- and ZOL-1-treated tumors showed a significant decrease in tumor volume, as well as in tumor cell density (Figure 3G). ZOL-1 treatment resulted in the elimination of GBM tumor cells, with only scarred stromal connective tissues remaining. These results suggest that the oncotropism of ZOL-1 observed in our in vitro experiments can be translated to an in vivo setting and can manifest as significant direct therapeutic benefits.

### 3.4. ZOL-1 Displays Effective Antitumor Action against Human GBM-Derived Cell Lines

**Figure 4:**
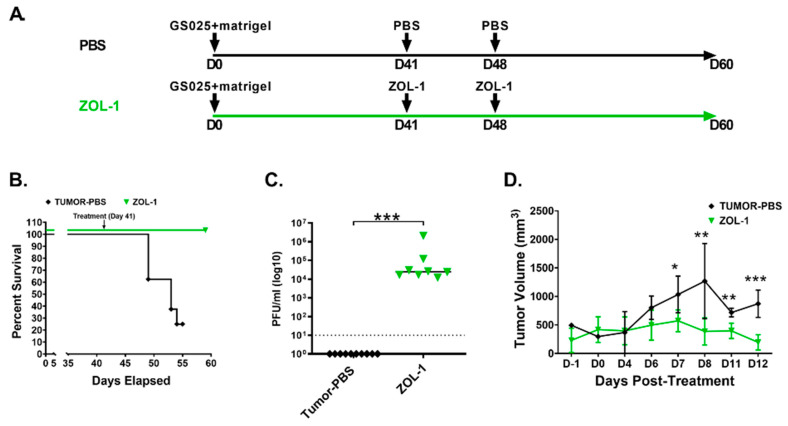
ZOL-1 therapeutic efficacy in vivo against human-derived tumors. (**A**) Experimental design of human-derived GBM (GS025) xenograft study with ZOL-1 treatment (n = 8 per group). (**B**) Kaplan–Meier survival plot shows percent survival of NSG mice upon mock treatment or ZOL-1 treatment. (**C**) Viral load of ZOL-1-treated mice at study endpoint (60 dpi). (**D**) Tumor volume measured in surviving animals at various timepoints post-xenograft injection. Error bars represent the standard deviation. Student’s *t*-test: *p*-value < 0.05 (*), *p* < 0.001 (**), *p* < 0.0001 (***).

After showing potential therapeutic efficacy in well-established U87 cells, we next performed an in vivo study using the human GBM-derived GS025 cell line, the responder cell line we analyzed in Figure 2. This cell line shows amplification of the EGFR receptor, which has been shown to promote resistance to chemotherapy and even treatment with EGFR inhibitors [59,60,61]. As such, we were interested in evaluating ZOL-1 as a prospective novel therapy in this chemo-resistant GBM line. A schematic of the study timeline can be found in Figure 4A. Mice were administered GS025 cells subcutaneously in the left-flank region (1 × 10^6^ cells/mouse). The development of palpable xenografted tumor was observed only a month post-cell engraftment compared with rapid tumor formation by U87 cells. On day 41, mice were treated with either ZOL-1 or PBS and were then administered another dose one week after primary treatment. Untreated mice succumbed to tumor burden and showed gradual increase in tumor volume, whereas all ZOL-1-treated mice survived and saw average decreases in tumor volume (Figure 4B,D). We were able to recover virus from ZOL-1-treated mice, suggesting direct antitumor effects resulting from treatment with ZOL-1 (Figure 4C). From these data, we see that the therapeutic benefit of ZOL-1 observed against U87 cells translated to human-derived chemo-resistant GS025 cells, with significant increases in survival and decreases in tumor volume.

### 3.5. Combination Therapies Enhance ZOL-1 Effects on Non-Responder GBM Cells

Active replication of ZIKV in non-responder lines with additional drug treatment can potentially manifest a combinatorial additive or synergistic effect, leading to a strong cell-killing response. The PI3K-AKT pathway has been extensively implicated in promoting carcinogenesis; cancer cell proliferation, invasion, metastasis, and drug resistance; and tumor angiogenesis [62,63]. As such, we wanted to assess the effect of PI3K-AKT-inhibiting compounds currently undergoing clinical trials combined with ZOL-1 on human-derived GBM apoptosis.

**Figure 5:**
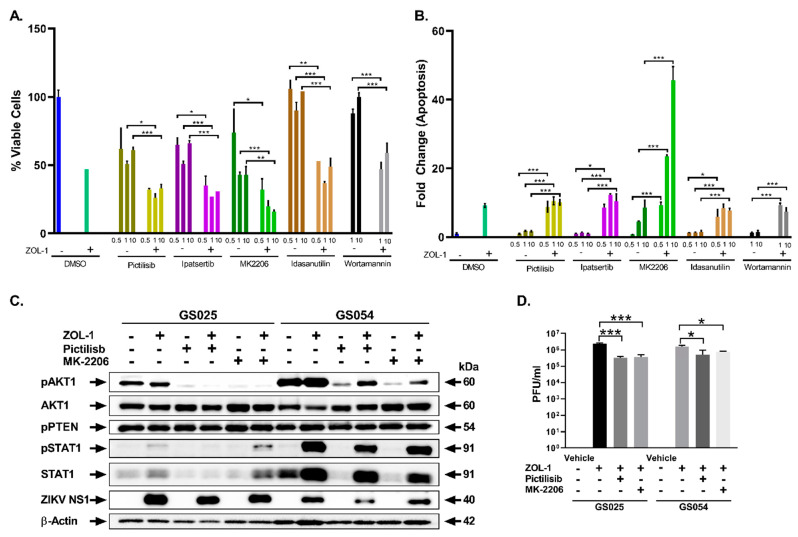
Assessment of PI3K-AKT pathway inhibitors with ZOL-1 combinatorial treatment. (**A**) Cell viability reduction of non-responder GS054 cells by various chemical AKT pathway inhibitors with and without ZOL-1 co-treatment. (**B**) Apoptotic cell death of non-responder GS054 cells induced by various chemical AKT pathway inhibitors with and without ZOL-1 co-treatment. (**C**) Western blot analysis of PI3K-AKT pathway intermediates in responder (GS025) and non-responder (GS054) cell lines upon treatment with pictilisib or MK2206 2HCl with or without ZOL-1 co-treatment. (**D**) ZOL-1 titer in responder and non-responder cell lines with or without treatment with pictilisib or MK2206 HCl. Student’s *t*-test: *p*-value < 0.05 (*), *p* < 0.001 (**), *p* < 0.0001 (***).

We tested the drugs pictilisib (inhibits class I PI3K isoforms [64]), ipatasertib (inhibits all three AKT isoforms [65]), MK2206 2HCl (allosteric inhibitor of AKT [66]), idasanutlin (inhibits MDM2 [67]), and wortmannin (irreversible inhibitor of the p110 PI3K subunit [54,68,69,70]). We observed that while co-treatment with ZOL-1 and each drug led to significant decreases in tumor cell viability and increases in apoptotic activity, MK2206 2HCl showed a much more drastic potentiation of cell death in non-responder cell line GS054 by ZOL-1 (Figure 5A,B). We did see an increase in cell killing in all other drug treatments when in combination with ZOL-1. However, the amount of caspase 3/7-mediated apoptotic cell death observed was comparable to that of ZOL-1 alone. Given that there was a reduction in viable cells, it is likely that cell death was facilitated by caspase 3/7-independent mechanisms or a direct inhibitory effect on cell-cycle proliferation. We then performed Western blot analysis 3 dpi with any combination of ZOL-1 and pictilisib or MK2206 2HCl upon treatment in both the responder GS025 line and the non-responder GS054 line (Figure 5C). The basal levels of pAKT1 were higher in the non-responder line compared with the responder line, suggesting higher activation of the PI3K-AKT pathway in this line. We also confirmed the inhibition of AKT phosphorylation upon drug treatment. Furthermore, to evaluate the impact of potential interactions between the drug candidates and ZOL-1, we assessed viral production in mock- and drug-treated groups using a viral plaque assay. While we observed a significant reduction in viral replication when utilizing combination treatment with drug and ZOL-1, this did not result in a deleterious reduction in viral load that would prevent therapeutic efficacy (Figure 5D). Based on these results, this experiment provides a case for a high-throughput combinatorial screening study evaluating other chemotherapies and kinase inhibitors alongside ZOL-1. Taken together, our study suggests the potential for developing personalized treatments based on patients’ tumor genotypic profiles.

## 4. Discussion

In this study, we demonstrated an effective therapeutic approach utilizing the attenuated ZIKV strain ZOL-1 against human-derived GBM cell lines. Treatment was shown to cause significant decreases in tumor volume upon lysis of tumor cells, resulting in potential tumor remission. We also dichotomized the cell lines assessed into responders and non-responders to ZOL-1 treatment, emphasizing the intertumoral heterogeneity of naturally occurring GBM tumors. Furthermore, when combined with various other chemotherapeutic and PI3K-AKT-inhibiting agents, we found that ZOL-1 shows potent antitumoral activity against human cell-derived neurospheres.

The concept of utilizing oncolytic viruses in treatment has been explored for nearly a century, with preclinical studies being published as early as 1949 [71,72]. More recently, researchers have attempted to utilize various neurotropic oncolytic viruses, such as herpes simplex virus [73], measles virus [74], and adenoviruses [75,76], alongside other conventional anticancer treatments, such as chemotherapies and radiotherapies.

The oncotropic and oncolytic behavior of ZIKV has been evaluated for years, with studies now suggesting its potential role as a therapy for GBM [77,78]. ZIKV has been shown to demonstrate potent oncolytic activity against GSCs, a property not shared by other neurotropic flaviviruses [44]. One common pitfall reported in past studies investigating the capability of ZIKV as an anti-GBM therapy has been a poor infection and replication rate in in vitro differentiated glioma cells, highlighting a commonly observed weak oncolytic effect on these cell types [41,45]. Our ZOL-1 construct showed robust cell infiltration and replication in the differentiated tumor cell types assessed while still displaying potent antitumoral effects (Figure 2B–D). Despite their observed weak oncolytic effect, studies still reported that use of an oncolytic ZIKV strain modified through a 10-nucleotide deletion in the 3′ untranslated region was highly safe—not targeting unaffected, normal brain cells—and established long-term immunological memory, which would prevent cancer recurrence [79]. Another study utilized a ZIKV strain modified through a point mutation in the NS4B gene that increased infectivity in GSCs [44].

It is known that tumors exist naturally as a widely heterogenous clonal mixture of cancer cells, cancer stem cells, and immune cells, among others [80,81]. As we demonstrated in our study, there are donor GBM cells that are more receptive to treatment than others. Interestingly, we found that the majority of non-responder lines had genetic changes in EGFR and PTEN (Table 1). At present, the genetic basis of the non-responder phenotype is not clear. As such, additional investigations are required to better understand this phenomenon.

In our in vivo studies, we found that ZOL-1 was highly effective in eradicating the implanted tumors. It is important to note that the mice used in this study were NSG mice and thus completely immune-naïve. In humans, we expect that treatment with ZOL-1 will also activate antitumor immune response, which will only further promote treatment efficacy. As gliomas and GBM are considered immunologically “cold” tumors, meaning that the cancer does not induce strong T-cell response [82], we expect that infiltration of cancer cells by ZOL-1 triggers directed antiviral immune response and thus converts an otherwise “cold” tumor into a “hot” tumor, or one that is targeted by directed immune response. However, this hypothesis requires further study in humanized in vivo models. The concept of converting a “cold” tumor into a “hot” tumor, especially GBM, has become increasingly studied. Overall, it has been concluded that treating most cancers, including GBM, is not often effective if only delivering one therapeutic target. By utilizing immune-stimulating and conventional arms of treatment, we hypothesize that GBM can be more readily treated and potentially complete tumor remission.

Given the heavy involvement of the AKT signaling pathway in GBM progression, we combined treatment with ZOL-1 with the use of AKT inhibitors. Many of the drugs utilized have been studied in phase II trials for various trials. Pictilisib has been shown to be effective in preclinical studies [83,84] and phase I trials against advanced solid tumors [85,86]. However, it was limited by dose toxicity in phase II trials for advanced breast cancer [87]. At the time of writing, no clinical trials for pictilisib have been conducted specifically for GBM. Ipatasertib and idasanutlin have successfully undergone phase I and II trials and are going through some phase III trials for various cancers, showing differing degrees of anticancer effects [88,89,90,91], and idasanutlin has demonstrated treatment efficacy in various GBM cell types [52]. Wortmannin has shown promise as a potential adjuvant to chemotherapy, though only in preclinical settings [92,93,94,95,96,97]. MK-2206 2HCl, which we found to be most effective in provoking antitumor response both in combination with ZOL-1 and alone, has been shown to have limited clinical activity in advanced breast cancer patients in a phase II trial [98]. As seen with pictilisib, this limited effect may be due to dose-toxicity-related concerns. In our drug study, we observed that despite these therapeutic limitations when used as a singular treatment, anticancer efficacy was increased when used as a combination therapy alongside ZOL-1. This direct inhibitory role against AKT, thus stifling the main proliferation pathway utilized by GBM cells, may explain why we observed such significant increases in antitumor activity in this treatment group.

Research on the therapeutic capacity of ZIKV mainly utilizes attenuated ZIKV platforms or recombinant ZIKV subunits [41,99,100], and questions still remain regarding overall safety and occurrence of adverse events. However, recombinant ZIKV has been shown to be safe with no apparent adverse events when utilized as a vaccine candidate [101]. We established ZOL-1 and AKT-inhibitor combination treatment to be safe and effective, as we observed no overt promotion or prevention of viral replication in combination-treated cells. Had we seen an overt promotion of viral replication, there may have been concerns of enhanced virulence of the ZOL-1 platform. A lack of viral replication would suggest that these drugs play an antagonistic role against ZOL-1, preventing its proliferation and treatment efficacy. Given that we saw no bias either way upon combination treatment alongside increased ZOL-1 treatment efficacy, we suggest that co-treatment of GBM cells with picitilisib or MK-2206 can enhance ZOL-1 oncolytic effects and establishes a proof of concept to further evaluate the role of AKT inhibitors in GBM treatment.

Taken together, these results present a strong case for the use of the safe and effective modified ZIKV construct, ZOL-1, as a part of a large-scale combinatorial screen with other chemotherapies and kinase inhibitors to develop novel methods to stimulate “cold” GBM tumors into “hot” sites that are more receptive to treatment. There is still work to be performed to establish the safety and efficacy of the ZOL-1 platform in GBM treatment, including conducting intracranial models in humanized immune system (HIS) mice, as well as assessing the impact of an intravenous route of administration, and the evaluation of non-responder lines with and without chemotherapeutic co-administration in vivo to better evaluate potential translation to human settings and assess immune cell infiltration of the tumor site. The development of this model can be highly useful for further clinical assessment and treatment modeling. Furthermore, given the variance in treatment receptivity that we observed across various GBM cell types, we also suggest that a personalized medicine approach, more specifically in vitro screens evaluating treatment efficacy in tumor eradication prior to the start of chemotherapy, may be a prudent step towards delivering maximally effective treatments for GBM.

## Figures and Tables

**Table 1:** Details of selected ZOL-1 responder and non-responder human-derived glioblastoma tumor cell lines.

Glioblastoma (GS) Sample ID *	Responder/Non-Responder	New/Recurrent	Ethnicity	Sex (M/F)	Age	EGFR CNV	PTEN CNV	PTEN MUT
GS054	Non-Responder	Recurrent	White	F	59		Shallow Deletion	G44D
GS122 **	Non-Responder	Recurrent	White	M	40	Amplified	Shallow Deletion	
GS147 ***	Non-Responder	Recurrent	White	M	59	Amplified	Shallow Deletion	
GS154	Non-Responder	Recurrent	Latino	M	51	Copy Number Gain		
GS194 ^+^	Non-Responder	Recurrent	White	M	52	Amplified	Shallow Deletion	
GS023	Responder	New	Mexican	F	47			X342_splice
GS025	Responder	Recurrent	White	F	39	Amplified		
GS118 ^+^	Responder	Recurrent	White	M	74		Shallow Deletion	
GS152 ^&^	Responder	New	Black	M	61		Shallow Deletion	N292Kfs*6
GS130 ^$^	Responder	Recurrent	White	F	61		Shallow Deletion	X70_splice

* All cases diagnosed were glioblastoma, IDH-wildtype; ** GS122 had detected EGFR vIII expression; *** GS147 had a PIK3CA mutation (E39K); ^+^ GS194 (A289V) and GS118 (R108K) had EGFR mutations; ^&^ GS152 tumor line was xenografted into mice to establish the GBX1152 tumor line; ^$^ GS130 tumor line was xenografted into mice to establish the XDS4130 tumor line.

## Data Availability

All relevant data regarding this manuscript are available from the above-listed authors.

## Supplementary Materials

The following supporting information can be downloaded at: https://www.mdpi.com/article/10.3390/cells12192384/s1, Table S1: Antibodies used in this study, Supplemental Figure S1: Visualization of ZOL-1 infection of glioblastoma cells. Bright-field images of mock- and ZOL-1 (MOI 1 and 10)-inoculated human-derived responder (GS118) and non-responder (GS122) tumor lines 7 dpi. (Scale bar: 50 µm.) Note: The GS122 line exhibited extreme sensitivity to 0.001% serum (FBS) present in the viral inoculum. At MOI 10, the cells differentiated to a monolayer culture; Supplemental Figure S2: Analysis of tumor size and ZOL-1 infiltration at study endpoint. (**A**) Representative images of tumors from mock- and ZOL-1-inoculated mice. (**B**) Immunofluorescence analysis of ZOL-1-mediated apoptosis of GBM cells. CC3: cleaved caspase 3; Env: ZIKV Envelope protein. (Scale bar: 25 µm.)
