# Supplementary figures and images for: Differential Susceptibility of Ex Vivo Primary Glioblastoma Tumors to Oncolytic Effect of Modified Zika Virus
Source: Cells. 2023 Sep 29;12(19):2384. doi: 10.3390/cells12192384 (PMC10572118; doi:10.3390/cells12192384)

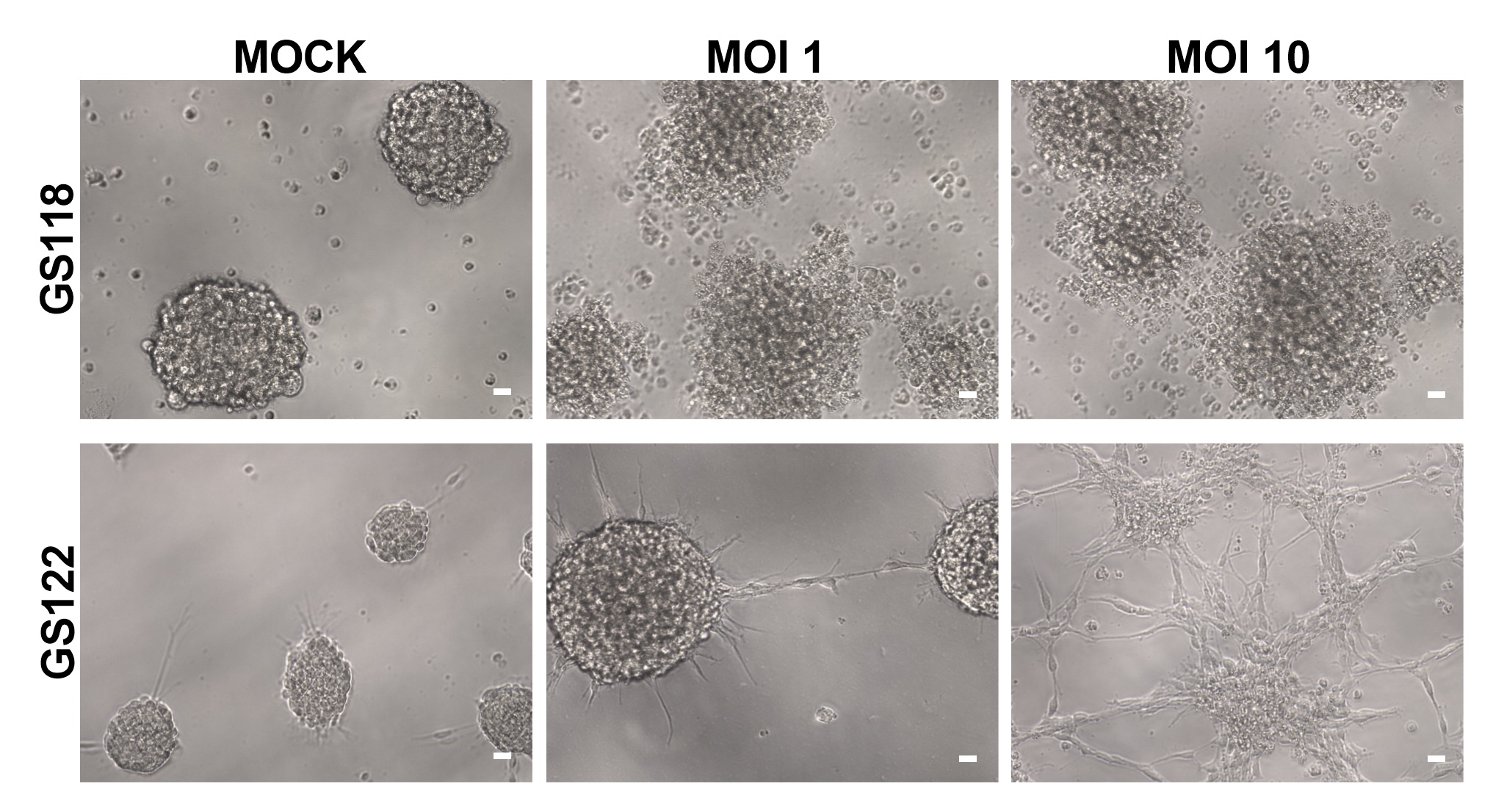

Supplement: Supplementary file 1 [file cells-12-02384-s001.zip › cells-2603892- 2 final supplementary/Supplemental Figures/Supplemental Figure 1_rev.jpg]

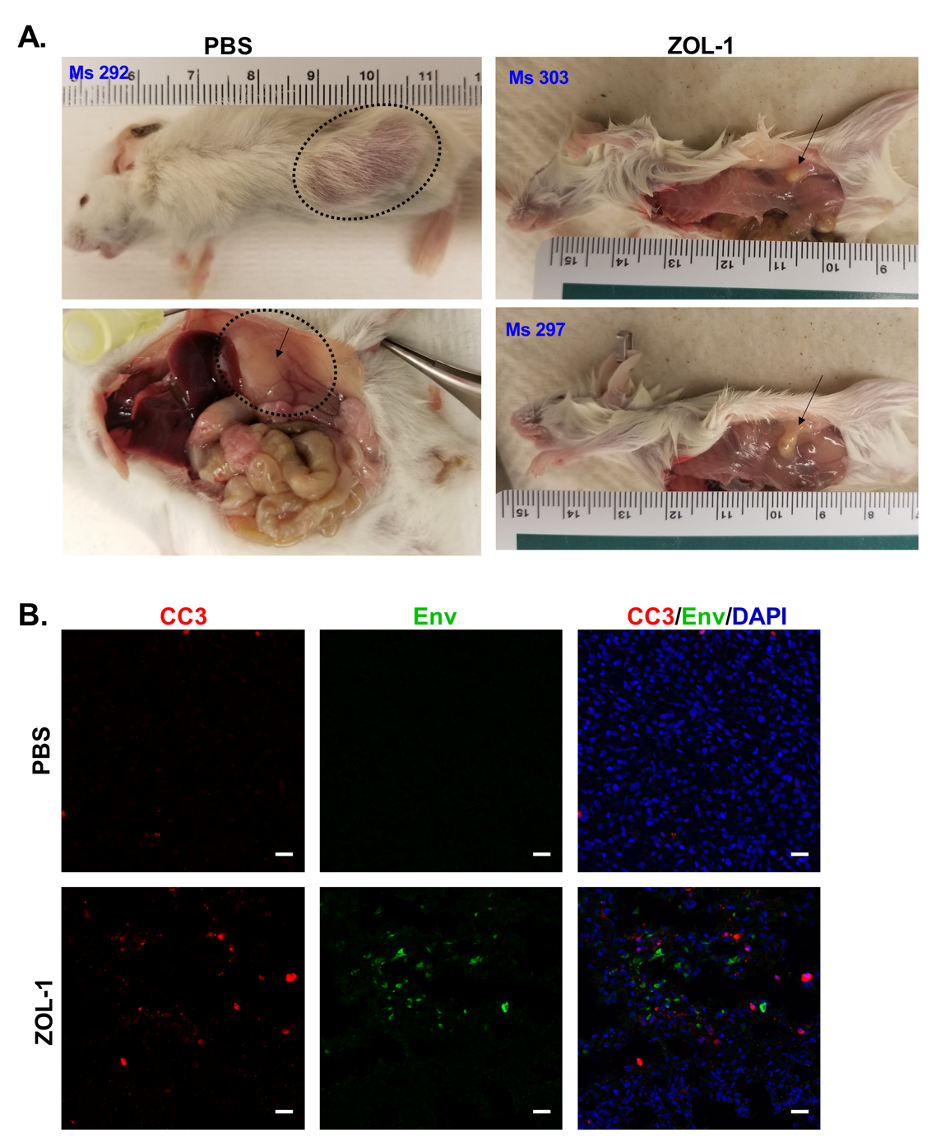

Supplement: Supplementary file 1 [file cells-12-02384-s001.zip › cells-2603892- 2 final supplementary/Supplemental Figures/Supplemental Figure 2.png]

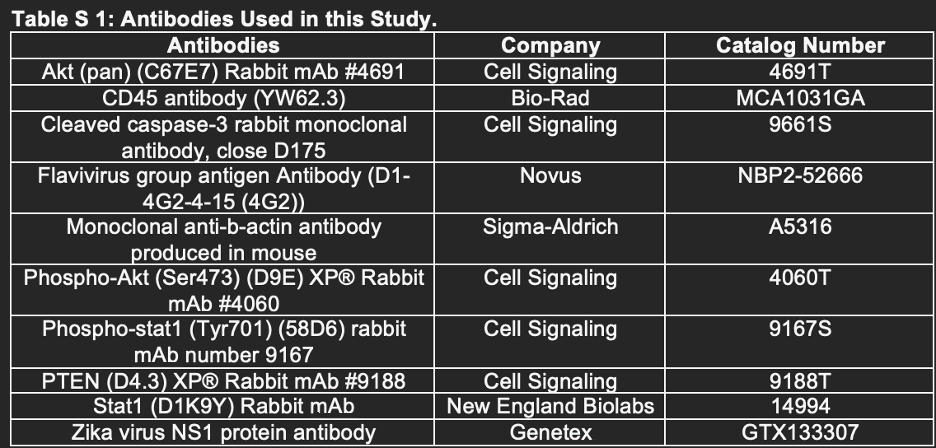

Supplement: Supplementary file 1 [file cells-12-02384-s001.zip › cells-2603892- 2 final supplementary/Supplemental Figures/Supplemental Table 1.png]
